# Metformin combined with spironolactone vs. metformin alone in polycystic ovary syndrome: a meta-analysis

**DOI:** 10.3389/fendo.2023.1223768

**Published:** 2023-08-10

**Authors:** Huamin Zeng, Ying Zhang, Sikai Huang, Jinghua Wu, Wenrui Ren, Lingru Zhou, Leneng Huang, Yuyin Ye

**Affiliations:** ^1^ Department of Endocrinology and Metabolism, The Third Affiliate Hospital of Guangzhou Medical University, Guangzhou, China; ^2^ Key Laboratory for Major Obstetric Diseases of Guangdong Higher Education Institutes, Guangzhou Medical University, Guangzhou, China; ^3^ The First Clinical School, Guangzhou Medical University, Guangzhou, China; ^4^ Paediatrics School, Guangzhou Medical University, Guangzhou, China

**Keywords:** polycystic ovary syndrome, metformin, spironolactone, combination, meta-analysis

## Abstract

**Aims:**

Due to its high heterogenicity and unclear etiology, there is currently no specific treatment for polycystic ovary syndrome (PCOS). Metformin, as an insulin sensitizer, combined with spironolactone, an antiandrogen medication, may exert complementary effects on PCOS. We therefore performed a meta-analysis of trials in which metformin combined with spironolactone was applied to treat PCOS to evaluate the efficacy and safety of the combination therapy.

**Methods:**

We retrieved the PubMed, Embase, Scopus, Cochrane Library, CNKI, CBM, Wangfang, and VIP databases for literatures published from their inception to December 16, 2022 on the effects of metformin combined with spironolactone in the treatment of PCOS. Inclusion criteria according to P.I.C.O.S criteria were: PCOS patients, metformin combined with spironolactone interventions, metformin alone control group, and randomized controlled trials with the following outcome data: body mass index (BMI), hirsutism score, luteinizing hormone (LH), follicle-stimulating hormone (FSH), total testosterone (TT), fasting blood glucose (FBG), Homeostatic Model Assessment for Insulin Resistance (HOMA-IR), and side effects including nausea, vomiting, diarrhea and drug withdrawal.

**Results:**

Our results revealed that metformin combined with spironolactone significantly reduced BMI and TT, but that it exerted no significant effects on hirsutism score, or on FSH or LH concentrations. Combined treatment also resulted in a significant diminution in FBG and insulin resistance using the HOMA-IR when the interventional time was greater than 6 months. In addition, the combination did not have a higher occurrence of adverse reactions than metformin alone.

**Conclusion:**

Compared with metformin alone, metformin combined with spironolactone therapy may be more effective in reducing BMI and serum androgen levels, but the combination showed no significant effect on the hirsutism score or gonadotropin hormone levels, and was not associated with an elevation in side-effects. Moreover, when the treatment course was greater than 6 months, combination therapy reduced FBG and improved insulin resistance more effectively than metformin alone. However, more research is needed to determine the most effective course of treatment.

**Systematic review registration:**

https://www.crd.york.ac.uk/PROSPERO/, identifier CRD42022355515.

## 1 Introduction

Polycystic ovary syndrome (PCOS) is a usual endocrine disease, affecting 5–20% of child-bearing period women. As the incidence of obesity has increased, the prevalence of PCOS is also increasing (1, 2). The Rotterdam diagnostic criteria are the most commonly used diagnostic criteria for PCOS, which must exclude the other causes and have at least 2 of the following three standards: 1) oligovulatory or anovulatory; 2) clinical or biochemical hyperandrogenism; and 3) polycystic ovaries under ultrasound (3). Due to an imbalance in female sex hormones, PCOS presents with a myriad of symptoms that include irregular menstrual cycles, anovulation, and hyperandrogenism. In addition to these classic symptoms, PCOS can also lead to metabolic disorders, including hypertension, cardiovascular issues, dyslipidemia, and increase the risk of endometrial cancer (4). Furthermore, previous study have shown that due to obesity, hirsutism and the decline in the quality of life, women with PCOS are at increased risk for anxiety and depression, and even more severe symptoms such as obsessive-compulsive disorder and somatization (5, 6).

As a result of the high heterogenicity of PCOS and unclear underlying cause(s), the treatment of PCOS is often symptomatic and individualized. At present, therapeutic options for PCOS range from pharmacologic treatments to surgery. Spironolactone, an androgen receptor blocker, plays an important role in the treatment of hyperandrogenism (7). Metformin, an anti-hyperglycemic agent, improves insulin resistance and enhances insulin sensitivity (8), has been proven to have a definite therapeutic effect on PCOS (9). Moreover, metformin has been also shown to be an effective treatment for pregnancy complications and obesity; it also improves menstrual cyclicity, but it is not effective for treating hirsutism (10). Some guidelines and reviews do not specifically describe the combination of metformin and spironolactone except when combined with other drugs such as combined oral contraceptive pills (COCPs) and clomiphene citrate (CC) (11).

In summary, when metformin is combined with spironolactone, a complementary effect may occur that better improves symptoms in PCOS patients. Using a meta-analysis, we herein summarized the current literature regarding the combined use of metformin with spironolactone in the treatment of PCOS, and examined the efficacy and drug safety of metformin combined with spironolactone when compared with metformin monotherapy in the treatment of PCOS.

## 2 Materials and methods

### 2.1 Data sources

#### 2.1.1 Information sources

Two investigators searched the PubMed, Embase, Scopus, Cochrane Library, CNKI, CBM, Wangfang, and VIP databases for literatures published from their inception to December 16, 2022. The keywords we use were as follows: “polycystic ovary syndrome”, “PCOS”, “Metformin”, “Spironolactone”. Using a combination of MeSH and text words, the specific search strategy we used in PubMed was as follows: (((((((((metformin[MeSH Terms]) OR (Dimethylbiguanidine[Title/Abstract])) OR (Dimethylguanylguanidine[Title/Abstract])) OR (Glucophage[Title/Abstract])) OR (Metformin Hydrochloride[Title/Abstract])) OR (Hydrochloride, Metformin[Title/Abstract])) OR (Metformin HCl[Title/Abstract])) OR (HCl, Metformin[Title/Abstract])) AND (((((((((((((((((((((((((((((((((Spironolactone[MeSH Terms]) OR (Spirolactone[Title/Abstract])) OR (Veroshpiron[Title/Abstract])) OR (Verospirone[Title/Abstract])) OR (Spiractin[Title/Abstract])) OR (Spirobeta[Title/Abstract])) OR (Spirogamma[Title/Abstract])) OR (Spirolang[Title/Abstract])) OR (Spirono-Isis[Title/Abstract])) OR (Spirono Isis[Title/Abstract])) OR (Spironone[Title/Abstract])) OR (Spirospare[Title/Abstract])) OR (Aldactone[Title/Abstract])) OR (Verospiron[Title/Abstract])) OR (Aldactone A[Title/Abstract])) OR (Aquareduct[Title/Abstract])) OR (Duraspiron[Title/Abstract])) OR (Espironolactona Alter[Title/Abstract])) OR (Espironolactona Mundogen[Title/Abstract])) OR (Flumach[Title/Abstract])) OR (Frumikal[Title/Abstract])) OR (Jenaspiron[Title/Abstract])) OR (Novo-Spiroton[Title/Abstract])) OR (Novo Spiroton[Title/Abstract])) OR (NovoSpiroton[Title/Abstract])) OR (Practon[Title/Abstract])) OR (SC-9420[Title/Abstract])) OR (SC 9420[Title/Abstract])) OR (SC9420[Title/Abstract])) OR (Spiro L.U.T.[Title/Abstract])) OR (Spiro Von Ct[Title/Abstract])) OR (Ct, Spiro Von[Title/Abstract])) OR (Von Ct, Spiro[Title/Abstract]))) AND ((((((((((((((((Polycystic Ovary Syndrome[MeSH Terms]) OR (Ovary Syndrome, Polycystic[Title/Abstract])) OR (Syndrome, Polycystic Ovary[Title/Abstract])) OR (Stein-Leventhal Syndrome[Title/Abstract])) OR (Stein Leventhal Syndrome[Title/Abstract])) OR (Syndrome, Stein-Leventhal[Title/Abstract])) OR (Sclerocystic Ovarian Degeneration[Title/Abstract])) OR (Ovarian Degeneration, Sclerocystic[Title/Abstract])) OR (Sclerocystic Ovary Syndrome[Title/Abstract])) OR (Polycystic Ovarian Syndrome[Title/Abstract])) OR (Ovarian Syndrome, Polycystic[Title/Abstract])) OR (Polycystic Ovary Syndrome 1[Title/Abstract])) OR (Sclerocystic Ovaries[Title/Abstract])) OR (Ovary, Sclerocystic[Title/Abstract])) OR (Sclerocystic Ovary[Title/Abstract])) OR (PCOS[Title/Abstract])).

#### 2.1.2 Inclusion and exclusion criteria

The inclusion criteria according to P.I.C.O.S criteria: (a) patients with PCOS were diagnosed using standard criteria; (b)metformin combined with spironolactone interventions;(c) metformin alone control group; (d) relevant and complete data were provided; (e) randomized controlled trials (RCTs).

The exclusion criteria: (a) where the control treatment for PCOS was spironolactone or the combination therapy was metformin combined with other treatments; (b) no relevant outcome indicators; (c) non-randomized controlled trials (NRCTs), duplicate studies, case reports, cell or animal studies and other non-clinical controlled trials.

### 2.2 Data extraction

Two evaluators (HMZ and SKH) independently used Microsoft Excel to extract relevant data as follows: Basic information (e.g., sex, age, and country); intervention measures; control measures; BMI; hirsutism score; hormonal status (i.e., levels of FSH, LH, and total T); metabolic parameters such as fasting blood glucose and HOMA-IR); and medication side-effects including nausea, vomiting, diarrhea, and the need for drug withdrawal. All discrepancies will reach a consensus through negotiation.

### 2.3 Missing data

We would contact the corresponding author via email to obtain data to complete the missing data in the included literature. If still not available, the literature would be excluded.

### 2.4 Data items

The variables we had abstracted from including study were as follows: population (sample, age, ethnicity, diagnostic criteria); intervention and comparison (type, dose, frequency, cointervention); outcomes (BMI; hirsutism score; hormone status (FSH, LH, and total T); metabolic parameters (FBG and HOMA-IR); adverse effects (nausea, vomiting, diarrhea, and the need for drug withdrawal)).

### 2.5 Measures of treatment effects

Continuous data are represented by a mean difference (MD) or standardized mean difference (SMD), binary data are represented by odds ratio (OR), and a 95% confidence interval (CI) was used for effect size estimation. We kept the units consistent of measuring data for each RCT.

### 2.6 Quality assessment

Two reviewers (SKH and YYY) independently used the Cochrane Collaboration tool and the Jadad scale evaluate the quality of the included literatures. Each risk of bias (selection, performance, detection, churn, reporting, and other bias) was assessed as high, low, and unclear using the Cochrane collaboration tool (12). Three items on the Jadad scale were randomized (2 points), blinded (2 points), and dropped (1 point) to assess the methodological quality of each study. The total score ≤2 was classified as low-quality experiment, and the total score ≥3 was classified as high-quality experiment.

### 2.7 Quantitative synthesis

We used ReviewManager (RevMan) V.5.3 to analyze the outcomes from including literatures. The effect size is the mean/standard mean (MD/SMD) OR odds ratio (OR) with their 95% CI. All data were measured using a fixed effect model. The I2 value and Q test in the forest plots were used to analyze the heterogeneity of the included literatures. When I2 is greater than 50%, the heterogeneity is considered to be high. Subgroup sensitivity analyses were performed and one study was sequentially excluded per iteration to understand their impact on the results. We used Stata 12.0 for sensitivity analysis, and the difference was statistically significant when p < 0.05.

### 2.8 Registration trial

Two reviewers(YYY and LNH) submitted a research proposal to the PROSPERO(International Prospective Register of Systematic Reviews, PROSPERO)and applied for a registration code (PROSPERO CRD42022355515).

## 3 Results

### 3.1 Literature search

**Figure 1:**
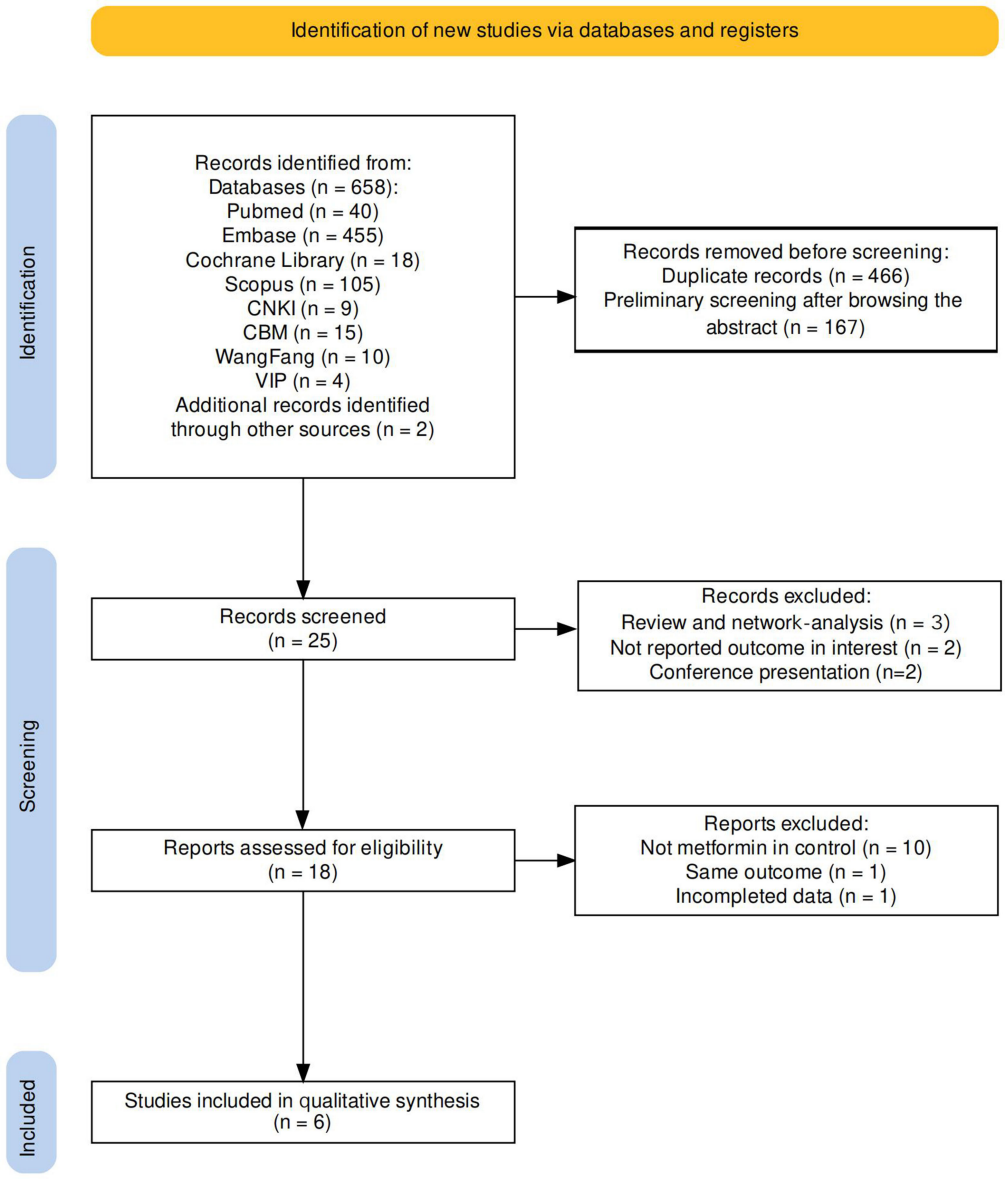
The flow chart of literature search is as follows.

The systematic research totally obtained 658 pertinent literatures (PubMed 40, Embase 45, Cochrane Library 18, Scopus 105, CNKI 9, CBM 15, Wangfang 10, VIP 4, another database 2). By eliminating duplicate articles and preliminary screening of literatures, 25 literatures remained. After excluding conference presentations (n = 2), review and network-analysis (n = 3), and studies that did not report outcomes of interest (n = 2), seven articles were removed. Among the 18 articles, we further excluded 12 articles that contained other drug treatments such as SPIOMET (a combination of spironolactone, pioglitazone, and metformin), where the control was not metformin therapy, and where the studies had incomplete data. Six articles were ultimately retained (13–18). The screen process is detail in **Figure 1** (19).

### 3.2 Quality assessment

**Figure 2:**
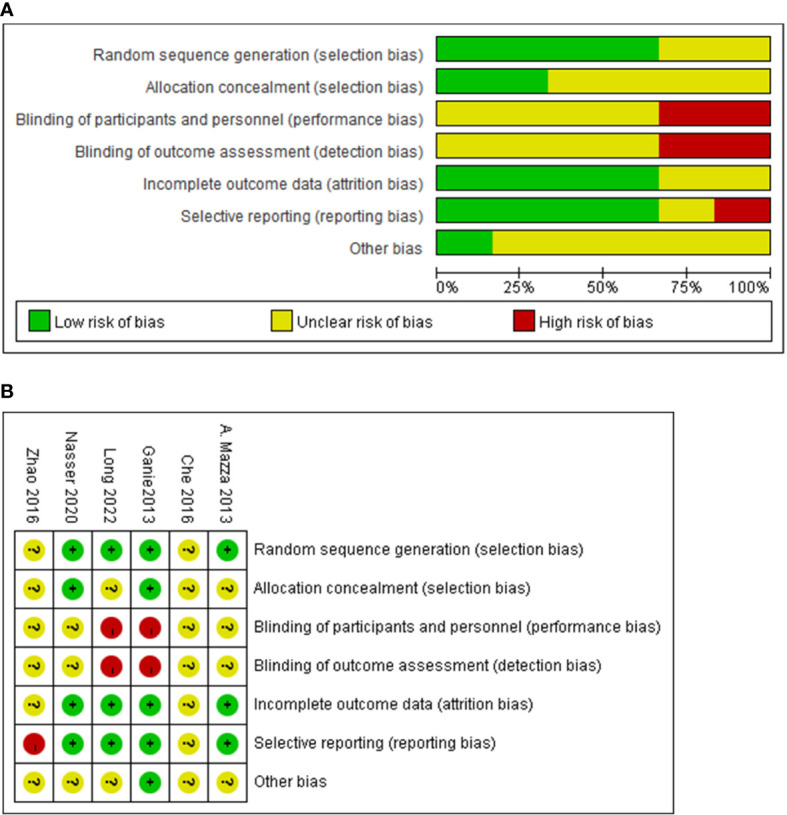
The quality of including studies by the Cochrane Collaboration tool. Summary of the risk of bias assessment **(A)**, and risk of bias **(B)**.

The quality assessment applying the Cochrane Collaboration tool was as follows: random sequence generation was assessed as low risk in four articles (13–16), whereas allocation concealment was described in only two articles (13, 16). An elevated risk of blinding of participants and personnel and blinding of the outcome assessment appeared in two open-label RCTs (15, 16). Attrition bias and reporting bias were of low risk in four articles. However, reporting bias was noted to be of high risk in one article since the results of the report were inconsistent with the data and could therefore influence the significance of our results (17). The risk of other biases was mostly assessed as unclear due to insufficient information provided. **Figure 2** illustrates the results of the risk-bias assessment.

Using Jadad scale, the total score of including studies was 1 to 4. Four studies were high-quality, but the low-quality studies were both from China. The Jadad scores of the included studies are displayed in **Table 1**.

**Table 1:** Summary of study characteristics and Jadad score for quality assessment of included studies.

First author and year of publication	Country	Diagnostic criteria	Study duration including follow-up(month)	Sample size			Age(year)	Experimental	Control	Conintervention	Jadad score
				Total	Intervention	control		Dose / Frequency	Dose / Frequency		
A.Mazza 2014	Italy	The Rotterdam Diagnostic Criteria	6	71	26	26	–	First week: Metformin 425mg/ twice daily Spironolactone 25mg/ once daily	First week: Metformin 425mg/ twice daily	Diet	3
								After first week: Metformin 825mg/ twice daily Spironolactone 25mg/ once daily	After first week: Metformin 825mg/ twice daily		
Che 2016	China	The Rotterdam Diagnostic Criteria	6	90	45	45	–	Metformin 500mg/ three times daily Spironolactone 20mg/ once daily	Metformin 500mg/ three times daily	–	1
Ganie 2013	India	The 2006 Androgen Excess Society Criteria	6	240	62	107	14∼39	Metformin 500mg/twice daily Spironolactone 20mg/ once daily	Metformin 500mg/twice daily	Diet + exercise	4
Long 2020	China	The Rotterdam Diagnostic Criteria	3	208	51	107	–	Metformin 1500mg/once daily Spironolactone 40mg/ once daily	Metformin 1500mg/once daily	Diet + exercise	3
Nasser 2020	Egypt	The Rotterdam Revised Criteria	6	48	22	21	20∼28	First week: Metformin 500mg/ twice daily Spironolactone 25mg/ once daily	First week: Metformin 500mg/ twice daily	Diet	4
								After first week: Metformin 850mg/ twice daily Spironolactone 25mg/ once daily	After first week: Metformin 850mg/ twice daily		
Zhao 2016	China	The Rotterdam Diagnostic Criteria	3	60	30	30	25~35	Metformin 1000mg/once daily Spironolactone 40mg/ once daily	Metformin 1000mg/once daily	Barrier method of contraception	1

### 3.3 Study characteristics

The principal characteristics of the six trials are presented in the Table below. In six clinical trials, a total of 717 patients, aged 14 to 40 years, were randomly assigned to combination therapy (n=236) and dimethylidyne (n=232), from different countries (China n=358, India n=240, Italy n=71, Egypt n=48). The duration of treatment maintained in clinical trials ranged from 3-12 months (mean 5 months, median 6 months), and most also combined a life intervention (diet/exercise). The dosage of metformin in clinical trials ranged from 850 to 2000 mg (median 1250 mg) and spironolactone ranged from 20 to 100 mg (median 32.5 mg). The diagnosis of PCOS is based on the Rotterdam and ESHRE criteria (**Table 1**).

### 3.4 Primary outcome

#### 3.4.1 The effect of drug combination on BMI

**Figure 3:**
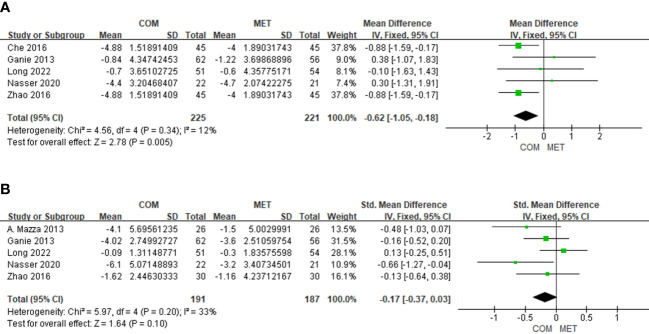
The forest plots of meta-analysis of BMI **(A)** and Hirsutism score **(B)**.

Five studies showed BMI changes relative to combination treatment and included 446 patients. The forest plot indicated that the combination treatment was superior to the metformin alone in BMI (MD, −0.62; 95% Cl, −1.05 to 0.18; P = 0.005); and heterogeneity in BMI was low (I^2 = ^12%) (**Figure 3A**).

#### 3.4.2 The effect of drug combination on hirsutism score

The results of hirsutism score changes relative to combination treatment in five studies comprised 378 patients. The modified Ferriman–Gallwey score (mFGS) and Ferriman–Gallwey score (FGS) represent the hirsutism score. We noted no significant difference in hirsutism scores (SMD, −0.17; 95% Cl, −0.37 to −0.03; P = 0.10), and the heterogeneity in the hirsutism scores was low (I^2 = ^33%) (**Figure 3B**).

#### 3.4.3 The effect of drug combination on FSH

**Figure 4:**
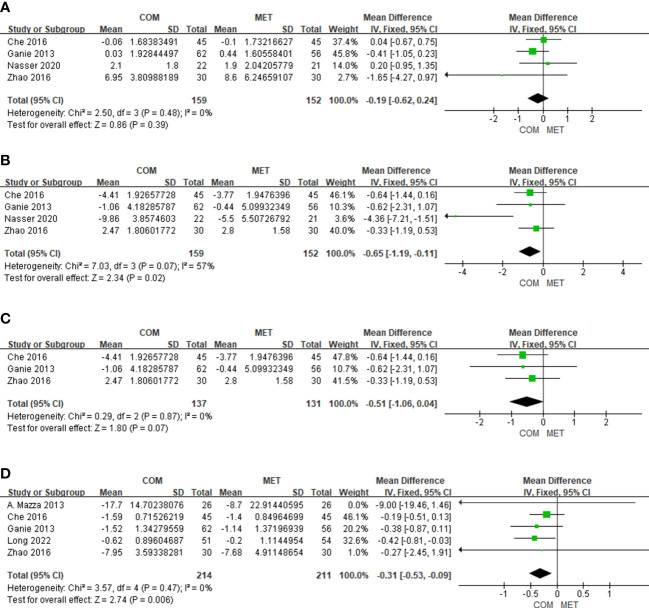
The forest plots of meta-analysis of FSH **(A)**, LH **(B)**, LH excluding Nasser 2020 **(C)**, and TT **(D)**.

The results of FSH changes relative to combination treatment were reported for 311 patients in four studies. There was no significant difference in FSH (MD, −0.19; 95% Cl, −0.62 to 0.24; P = 0.39), and heterogeneity in FSH was low (I^2 = ^0%) (**Figure 4A**).

#### 3.4.4 The effect of drug combination on LH

Four studies showed results of LH changes relative to combination treatment in 311 patients. For LH reduction, the combination treatment was more effectively than the metformin alone and was statistically significant (MD, −0.65; 95% Cl, −1.19 to −0.11; P = 0.02), while heterogeneity was slightly higher (I^2 = ^57%) (**Figure 4B**). When we excluded the study by Nasser published in 2020, the heterogeneity would decrease (I^2 = ^0%), and there was still significant difference in LH (**Figure 4C**). The most probable reason for the result is that the patients in the clinical trial by Nasser were overweight/obese.

#### 3.4.5 The effect of drug combination on TT

Five studies reported the results of TT changes relative to combination treatment included 425 patients (**Figure 4**). With respect to lowering TT, the forest plot showed that the combination was more effective than metformin alone with low heterogeneity (I^2 = ^0%) (**Figure 4D**).

#### 3.4.6 The effect of drug combination on FBG

**Figure 5:**
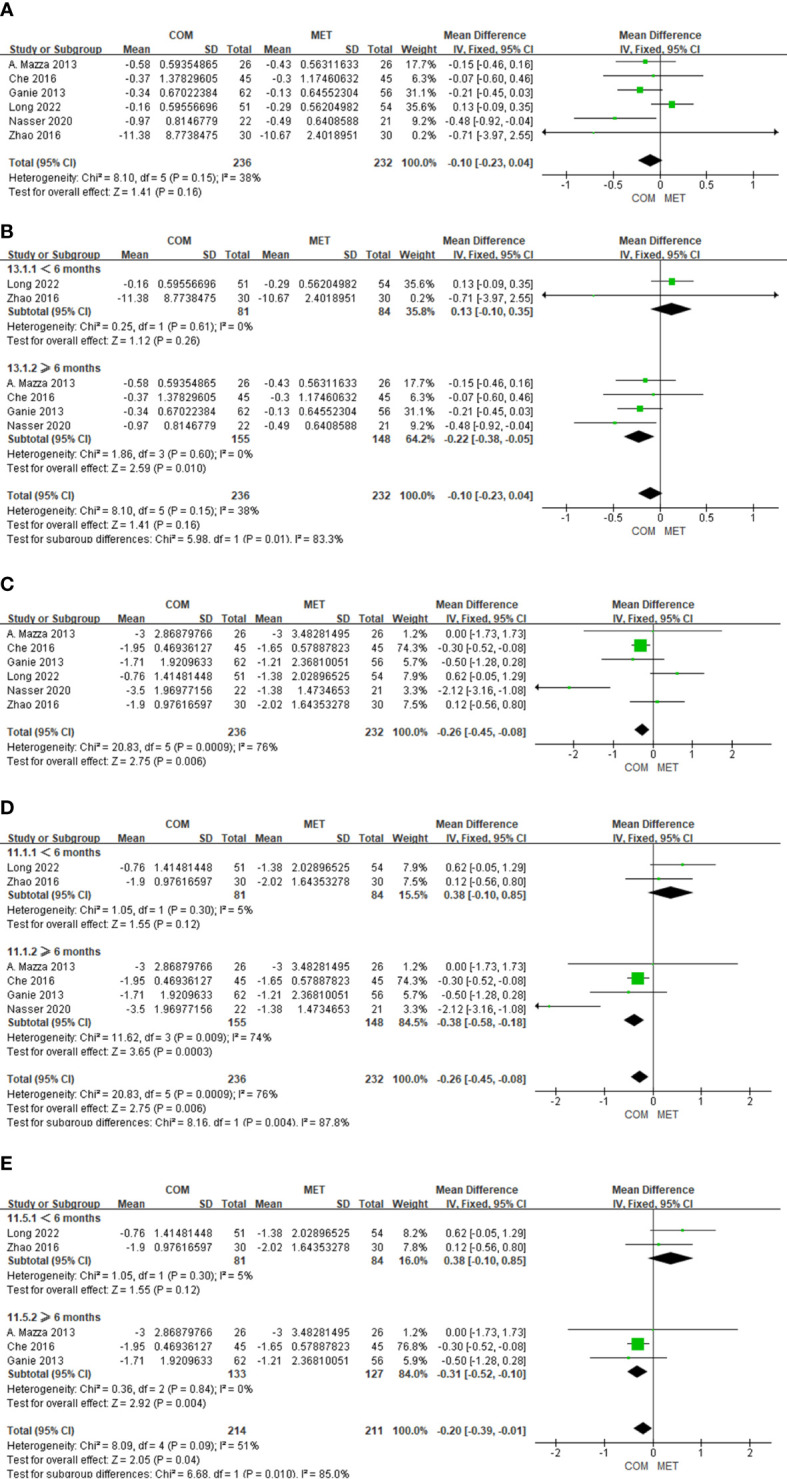
The forest plots of meta-analysis of FBG **(A)**, FBG according the study duration **(B)**, HOMA-IR **(C)**, HOMA-IR according the study duration **(D)**, and HOMA-IR excluding Nasser 2020 **(E)**.

Six studies described results of FBG changes relative to combination treatment and included 468 patients. Combination therapy of metformin and spironolactone did not significantly improve FBG levels (P = 0.16), with mild heterogeneity uncovered (I^2 = ^38%) (**Figure 5A**). We performed a subgroup analysis based on duration of treatment, and found that when intervention time was ≥6 months, the combination of metformin and spironolactone reduced FBG levels (MD, −0.22; 95% Cl, −0.38 to −0.05; P = 0.01) with low heterogeneity (I2 = 0%) (**Figure 5B**).

#### 3.4.7 The effect of drug combination on HOMA-IR

In six studies the results of HOMA-IR changes relative to combination treatment comprised 468 patients. We observed a significant difference in HOMA-IR (P = 0.006), with high heterogeneity detected (I^2 = ^76%) (**Figure 5C**). When we conducted subgroup analysis where the intervention time was < 6 months, combination therapy did not improve HOMA-IR status (P = 0.12), and there was low heterogeneity (I^2 = ^5%). However, when the intervention time was ≥ 6 months, combination therapy of metformin and spironolactone diminished HOMA-IR status (P = 0.0003), with high heterogeneity shown (I^2 = ^74%) (**Figure 5D**). Through sensitivity analysis, Nasser’s study showed significant heterogeneity compared with other studies. After removing Nasser’s study, heterogeneity decreased (I^2 = ^0%) and the overall effect still remained significantly different (P = 0.004) (**Figure 5E**). The high heterogeneity may be due to the fact that the subjects included in the Nasser’s trial were overweight or obese.

#### 3.4.8 The effect of drug combination on nausea

**Figure 6:**
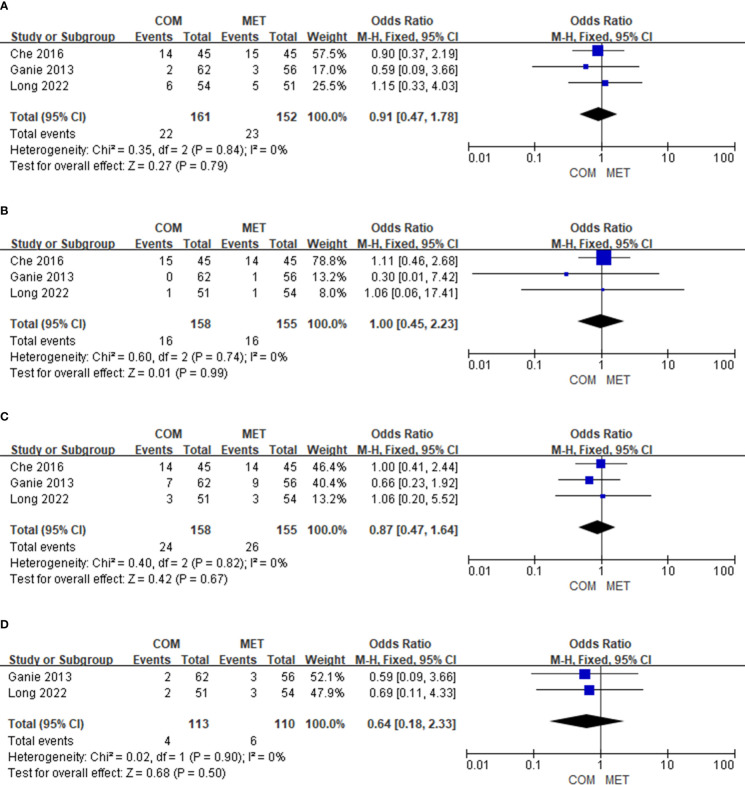
The forest plots of meta-analysis of nausea **(A)**, vomiting **(B)**, diarrhea **(C)**, and drug withdrawal **(D)**.

Three studies on the results of nausea events included 313 patients. There was no significant difference in nausea (OR, 0.95; 95% Cl, 0.48 to 1.85; P = 0.87), and heterogeneity in nausea was low (I^2 = ^0%) (**Figure 6A**).

#### 3.4.9 The effect of drug combination on vomiting

Three studies on vomiting events comprised 313 patients. There was no significant difference in vomiting (OR, 1.00; 95% Cl, 0.45 to 2.23; P = 0.99), and heterogeneity with respect to vomiting was low (I^2 = ^0%) (**Figure 6B**).

#### 3.4.10 The effect of drug combination on diarrhea

Three studies on diarrhea events included 313 patients. There was no significant difference in diarrhea (OR, 0. 87; 95% Cl, 0.47 to 1.64; P = 0.67), and heterogeneity in diarrhea was low (I^2 = ^0%) (**Figure 6C**).

#### 3.4.11 The effect of drug combination on drug withdrawal

Six studies depicted the results of drug withdrawal events and included 467 patients. The reason for drug withdrawal was primarily intolerance, with no other reasons given. There was no significant difference in drug withdrawal (OR, 0.64; 95% Cl, 0.18 to 2.33; P = 0.50), and heterogeneity was low (I^2 = ^0%). In addition, there were no reports of serious adverse effects such as hyperkalemia or of elevated levels of creatinine or urea nitrogen in the six included studies (**Figure 6D**).

## 4 Discussion

PCOS is an endocrine disease that can lead to metabolic syndrome and simultaneously a psychiatric disease that can harm women’s health and their quality of life. The etiology of PCOS, however, is not currently understood. International evidence-based guidelines propose the following factors as potential causes of PCOS: hypothalamic-pituitary dysfunction, insulin resistance and hyperinsulinemia, hyperandrogenism, abnormal ovarian regulatory mechanisms, genetic predisposition, and the environment (11). Insulin resistance can interfere with the hypothalamic-pituitary-ovarian axis, and hyperinsulinemia amplifies the androgenic potential of the ovarian theca cells and enhances the secretion of androgens (20). The increase in androgen sensitivity and utilization and the decrease in its clearance all contribute to the hyperandrogenism, which then further inhibits the development and ovulation of follicles and causes polycystic ovaries and anovulation (11, 21). Therefore, improving insulin resistance and treating hyperandrogenemia constitute the focus of treatment for PCOS.

Metformin is an insulin sensitizer that can decrease glucose absorption in the gut lumen, increase glucose uptake and utilization by peripheral tissues, inhibit glucose production, improve insulin resistance, reduce body weight, and achieve euglycemia. It is the first-line treatment for type 2 diabetes mellitus (T2DM) and has also been shown to be effective in the treatment of PCOS (9). Spironolactone is an androgen receptor blocker, exerts antiandrogenic effects directly by blocking androgen receptors, stimulates aromatase, and partially blocks androgen synthesis (7). In addition, spironolactone is able to inhibit inflammation by preventing aldosterone from binding to mineralocorticoid receptors, and its positive effect on insulin resistance has also been demonstrated in clinical and experimental studies (22). Diamanti-Kandarakis et al. (23) demonstrated that metformin improved hirsutism, and Ganie et al.^15^ showed that spironolactone improved insulin sensitivity, although to a lesser degree than metformin. Adeyanju et al. (24) also found that spironolactone improved IR in PCOS patients, most likely by suppressing the elevations in T, and that spironolactone exerted a protective effect. The combination of the two drugs in theory, then, should act in a complementary fashion.

Our study included six RCTs, four of which were of high quality (7). We ascertained that the combination of metformin and spironolactone was more beneficial to improve BMI and TT, but that it was not more effective on FGS, FSH, or LH than metformin alone. In addition, combination treatment resulted in a significant diminution in FBG and HOMA-IR when the duration of therapy was ≥ 6 months. However, in a meta-analysis of 28 RCTs that encompassed 1631 PCOS patients, Chen et al. determined that 1000 mg/day of metformin alone was needed for at least 25.5 weeks and that 1000 mg/day of metformin combination therapy was required for at least 58.6 weeks to produce better curative effects on body weight. The reason for the longer time period required to achieve effects by the combination therapy (e.g., metformin + simavastin, metformin + drospirenone/ethinyl estrdiol, metformin + ethinyl estradiol/norgestimate, metformin + flutamide, and metformin + rosiglitazone) might be the perceived synergistic actions between the two drugs, which are time-dependent. The reason for this may be that the weight gain caused by combination drugs, such as rosiglitazone and ethinylestradiol, counteracts the weight reduction effect of metformin, which prolongs the effective duration of weight reduction in combination therapy. These effects are also somewhat contradictory to the present study, which may be due to the dosages of metformin used (which ranged from 1000 to 1700 mg/day) in our included studies and the fact that insulin resistance (IR) changed earlier than BMI. In addition, previous studies have shown that IR resulting from abnormal insulin signaling and metabolic dysfunction in insulin-responsive tissue was elevated or that augmented IR was associated with increased weight gain (25–27). Similarly, Kirstin et al (28) investigated variations in insulin sensitivity and BMI and found that women with high BMI were at greater risk of impaired insulin sensitivity and elevated glucose levels during the luteal phase of their menstrual cycles. The findings of Song also suggested that a high IR resulted in low future weight gain because of a negative feedback mechanism by low glucose oxidation (26). Furthermore, Johnson (29) demonstrated in his 2014 analysis that both high BMI and insulin resistance contributed to the pathogenesis of PCOS. Therefore, metformin, as a common insulin sensitizer, was considered to be a priority option treatment for women with PCOS and insulin resistance. Noteworthy is that metformin exerts different effects on overweight/obese and non-obese women with PCOS. Yang et al. (30) indicated that HOMA-IR and insulin levels of overweight women decreased significantly after using metformin for 12 months and returned to the baseline levels for 24 months, whereas there was no discernible change in HOMA-IR and insulin levels in non-obese patients during 24 months. We interpret this to mean that the high heterogeneity of the Nasser study was due to the authors’ inclusion of overweight/obese patients. Unfortunately, for the present study we could not obtain the original BMI data for subgroup analysis in order to further prove the efficacy of metformin in obese and non-obese women with PCOS. In addition, compared with the hyperinsulinemic-euglycemic clamp test, the gold standard of assessing insulin resistance, HOMA-IR, still has several limitations and may affect the accuracy of the study results (31).

On the one hand, spironolactone could improve insulin resistance by lowering androgens, reduce body weight and BMI. In addition, Mineralocorticoid receptors (MR) activation has been shown to trigger abnormal responses in various tissues, including adipose tissue. Spironolactone, as an aldosterone antagonist, can block the activation of MR, reduce the expression of inflammatory bodies and the inflammation of adipose tissue, decrease the levels of various inflammatory indicators (NF-κB, TNF-α, IL-6), improve the oxidative stress and antioxidant capacity, and protect the injured adipose cells (32). In addition, spironolactone acts only on the distal tubules and collecting ducts as diuretics, resulting in a potassium-sparing diuretic effect, simultaneously reduction in plasma volume would activate the neurohumoral systems (the renin-aldosterone-angiotensin system, sympathetic nervous system, and ADH secretion), leading to a relative increase in Na and water absorption. Therefore, spironolactone has a weak diuretic effect and is less likely to reduce the patient’s body weight through Na and water excretion, which is not considered as the main reason for the reduction of BMI in combination therapy (22). In summary, spironolactone combined with metformin can reduce androgen, improve insulin resistance, promote the remission of lipolysis, and improve glucose uptake and energy homeostasis together with metformin, ultimately resulting in weight loss in patients. This may be the main mechanism of combination therapy.

Hyperandrogenism is characterized by the increase or over-activity of male hormones in the blood circulation, which can lead to the dysfunction of the hypothalamic-pituitary-ovarian axis and the imbalance of energy metabolism in women. Clinically, it mainly presents symptoms such as hirsutism, acne, obesity and irregular menstruation. Hirsutism can be quantified by the androgen level based on the hirsutism score (Ferriman-Gallwey/modified Ferriman-Gallwey) and is the most commonly used clinical diagnostic criterion for hyperandrogenism. Relevant study has shown that the severity of hirsutism is correlated with biochemical hyperandrogenism markers, and hirsutism score has a stronger correlation with FT level, but a lesser extent to TT level (33). It is speculated that the severity of hirsutism depends mainly on the bioavailable circulating and FT. This may also be the reason why the combination treatment in this study effectively reduced TT level, but did not significantly improve the hirsute score. Unfortunately, FT data could not be collected in this study.

It should be noted that both metformin and spironolactone have side-effects. Maliha et al. demonstrated that the use of metformin was related to a high incidence of side-effects, particularly gastrointestinal (GI) reactions such as nausea, vomiting, diarrhea, and abdominal pain. While the most serious adverse effect of metformin is lactic acidosis, it is quite rare (34). The side-effects of spironolactone include hyperkalemia, transient polyuria, gastrointestinal discomfort, nausea, breast tenderness, allergic reactions, somnolence, headache, vertigo, and menstrual irregularity (35). However, these side-effects are generally mild and rarely lead to drug withdrawal. Intriguingly, regardless of which drug is used, the side-effects of metformin and spironolactone are both closely dose-related (36). Metformin is generally administered at a dosage of 500–2000 mg/day, and the dosage for spironolactone ranges from 25 to 400 mg daily depending upon the specific disease. In order to avoid adverse drug reactions, the dosage of metformin or spironolactone at the initiation of treatment usually begins at a low level. In our study, none of the six included studies reported serious adverse reactions, including hyperkalemia or elevated levels of urea nitrogen or serum creatinine. In only three studies (15–17) did the investigators record adverse reactions (mainly gastrointestinal reactions), and in one study (16) metrorrhagia was reported. We also found that the incidence rates of these side-effects and of drug withdrawal were quite low, and that there was no significant difference between metformin alone and metformin combined with spironolactone. Therefore, metformin combined with spironolactone will not increase the occurrence of side-effects compared with metformin therapy alone, and the combination is safe and tolerated by patients. However, our sample of included studies was small and the dosage of spironolactone was low (20–50 mg/day); therefore, additional clinical studies are needed to verify the apparent side-effects of therapy with metformin combined with spironolactone.

There were some limitations to our meta-analysis. There were only six RCTs included, and the majority involved small sample sizes, high attrition rates, and poor patient adherence. Moreover, due to the limited number of included studies, publication bias was not assessed. In addition, the duration of treatment was limited to 3 to 6 months, and parameters of the menstrual cycle and blood lipid metabolism were lacking. Moreover, other factors may have produced clinical heterogenicity, such as ethnic groups, age, baseline BMI, the dosages of metformin and spironolactone, and dietary and exercise guidelines.

## 5 Conclusions

Compared with metformin alone, we ascertained that metformin combined with spironolactone therapy was more effective in reducing BMI and serum androgen levels, but it exerted no significant effects on hirsutism score or hormone levels and did not produce more side-effects. Moreover, when the course of treatment was ≥ 6 months, combination therapy lowered fasting blood glucose and improved insulin resistance more effectively than metformin; however, additional studies need to be conducted to determine the most effective course of treatment.

## Author contributions

Conception and design: HZ. Analysis and interpretation: HZ, SH, JW, WR, and LZ. Data collection: HZ, SH, and LZ, Article writing: HZ, SH, and LH. Critical revision of the article: HZ and SH. Final approval of the article: HZ, SH, JW, WR, LZ, LH, and YY. Statistical analysis: HZ, SH, JW, and WR. Overall responsibility: YZ.
